# Patterns and Characteristics of Diabetic Ketoacidosis in Children With Type I Diabetes in Saudi Arabia

**DOI:** 10.7759/cureus.55857

**Published:** 2024-03-09

**Authors:** Rasha Alradadi, Daliah M Alharbi, Maram S Alrehely, Samah F Alraddadi, May Almouteri, Muna Mohammad AlSuhaimi, Maram Abdullah Alaofi, Noha Farouk Tashkandi, Fatimah A Aljohani

**Affiliations:** 1 Pediatric Endocrinology, Taibah University, Medina, SAU; 2 College of Medicine, Taibah University, Medina, SAU; 3 Pharmacy, Medical Center at Taibah University, Medina, SAU; 4 Pharmaceutical Care, King Salman Medical City, Medina, SAU; 5 Medical Research, King Saud bin Abdulaziz University for Health Sciences, College of Medicine, Riyadh, SAU; 6 Pharmaceutical Care, Prince Sultan Armed Forces Hospital, Medina, SAU

**Keywords:** pediatrics, saudi arabia, retrospective chart review, diabetic ketoacidosis, type 1 diabetes mellitus

## Abstract

Background: Type 1 diabetes mellitus (T1DM) in children, a significant public health concern, often leads to diabetic ketoacidosis (DKA). The prevalence of T1DM is increasing globally, with Saudi Arabia recording high rates of DKA at T1DM onset. This study aimed to evaluate the characteristics and risk factors of pediatric T1DM patients presenting with DKA in the emergency room in Saudi Arabia and quantify intensive care unit (ICU) admission incidences reflecting DKA severity.

Methods: This retrospective chart review, conducted at Medina Maternity and Children's Hospital, Saudi Arabia, analyzed data from 2017 to 2022. The study included children and adolescents under 18 presenting with DKA, using non-probability consecutive sampling. Patient medical records provided demographic, medical, and laboratory data, and the analysis employed SPSS for statistical assessment.

Results: The study enrolled 70 participants, predominantly female (n = 42, 60%) and Saudi nationals (n = 63, 90%). The average age at diabetes mellitus (DM) onset was 6.9 years, with a mean hospital stay of 3.31 days. About 18.57% (n = 13) were newly diagnosed with DM, and 81.43% (n = 57) were known cases of DM. Most participants (n = 59, 86.8%) had no comorbidities, while 7.4% (n = 5) had celiac disease. The recovery rate was high (n = 67, 95.7%), with 80% (n = 56) experiencing no complications. Notably, 44.3% (n = 31) were admitted to a ward, and 12.9% (n = 9) required ICU admission. Weight was found to be a significant predictor of ICU admission (OR = 1.26, 95% CI: 1.05 to 1.5; p = 0.011).

Conclusions: This study highlights the importance of personalized insulin therapy and weight management in pediatric T1DM patients presenting with DKA. It suggests that early and effective management in emergency settings can significantly improve patient outcomes. The study also calls for further research into long-term management strategies and the impact of targeted educational programs.

## Introduction

Type 1 diabetes mellitus (T1DM) in children is a critical public health concern, marked by its chronic nature and the severe complications it can cause, including diabetic ketoacidosis (DKA) [1]. DKA, characterized by hyperglycemia, ketosis, and metabolic acidosis, represents a significant emergency in pediatric diabetes care [2]. The increasing prevalence of T1DM in children, along with the increasing instances of DKA, necessitates a deeper understanding of this complication's dynamics. Globally, over 96,000 children and adolescents below 15 years are diagnosed with T1DM every year [3], with 13% to 80% of them exhibiting DKA at diagnosis [4]. Notably, Saudi Arabia records one of the highest rates of DKA at T1DM onset, with a prevalence of 44.9% [5].

In Tabuk, Saudi Arabia, a retrospective study focusing on children under 12 years found that 38.0% (106 out of 279) presented with DKA [6]. The study noted that females, underweight children, and those in the 0-3-year age group were at the highest risk of developing DKA [6]. Additionally, a recent cross-sectional study of 103 adolescents with T1DM in Saudi Arabia's central region revealed a higher frequency of recurrent DKA in adolescents with elevated HbA1c levels, lipodystrophy, and a history of discontinuing insulin treatment [7]. The study underscored the need for comprehensive, multidisciplinary diabetes education to reduce modifiable risk factors in Saudi T1DM patients [7].

Several risk factors contribute to the development of DKA in children with T1DM. Insufficient diabetes education, poor adherence to insulin therapy, and delayed T1DM diagnosis are pivotal in precipitating DKA episodes [8]. Socioeconomic disparities further exacerbate this issue as access to healthcare and diabetes management resources remains a challenge for many families [9]. Demographic factors, including age and ethnicity, also influence the risk of DKA at T1DM onset, suggesting that personalized approaches to diabetes care might be beneficial [10].

This study aimed to evaluate the characteristics of children with T1DM who present to the emergency department (ED) and the risk factors of DKA. Additionally, we aimed to quantify the incidence of intensive care unit (ICU) admissions with a primary diagnosis of DKA, possibly reflecting the severity of these cases.

## Materials and methods

Study setting and design

This retrospective chart review was conducted at Medina Maternity and Children's Hospital, Saudi Arabia. The study collected patients’ data from 2017 to 2022, offering a retrospective view of the pediatric presentations of DKA over these years. The protocol of this study was approved by the King Salman Bin Abdulaziz Medical City Institutional Review Board (IRB number: 23-003).

Study participants

Participants were selected based on specific inclusion and exclusion criteria. The study included children and adolescents under 18 years who presented to the ED with DKA during the 2017-2022 timeframe. The diagnosis of DKA was confirmed based on the biochemical criteria established by the International Society for Pediatric and Adolescent Diabetes (ISPAD) [11], which includes hyperglycemia (blood glucose > 11 mmol/L or approximately 200 mg/dL), varying degrees of metabolic acidosis, and the presence of ketonemia and ketonuria.

Patients whose DKA was secondary to type 2 diabetes; those with comorbid conditions that influence ketone production, such as metabolic diseases; and cases with incomplete biochemical criteria for DKA were excluded from the study. Specifically, those lacking acidosis or those presenting with confounding conditions like chronic renal failure or cardiac disease were not considered for the study.

Sample size and sampling technique

The sample size was determined using a Chi-squared test, taking into account the results of a similar study conducted by Almalki et al. [12]. With an alpha of 0.05 and a power of 80%, the required sample size was established as 192 participants. The study employed a non-probability consecutive sampling technique. All diabetic children who presented to the ED during the study period and met the inclusion criteria were included in the sample.

Data collection methods and measurements

Data collection involved reviewing patient medical records using a specifically designed data collection sheet. The extracted data included demographic information such as age, gender, nationality, comorbidities, weight, height, and socioeconomic status. Medical information encompassed the type and duration of diabetes, presenting signs and symptoms, complications, duration of hospital stay, altered consciousness, frequency of DKA and mental obtundation, duration of DKA episodes, medications used for diabetes management, and insulin therapy details (type, dose, and adjustments). Laboratory data, including glycated hemoglobin (HbA1C) levels before the DKA episode, lactic acid, blood pH levels, and electrolytes (Na, K, Phos, arterial blood gas [ABG], or venous blood gas [VBG]), were also recorded. Patient outcomes, specifically the development of complications or mortality, were noted.

Data management and analysis plan

Data were entered and analyzed using the Statistical Package for Social Science (IBM Corp., Armonk, NY). Qualitative variables were presented as frequency and percentages, while quantitative variables were depicted as mean and standard deviation. Descriptive data on associated causes and risk factors were presented, and the significance of relationships between risk factors and other demographic and clinical factors was assessed using the Chi-square test, independent T-test, or Mann-Whitney test. The level of significance was set at 0.05. Additional inferential statistics were employed as appropriate based on variable types, and Odds ratios with 95% confidence intervals were used to evaluate the predictors of ICU admission.

## Results

Demographic and clinical characteristics

The study enrolled 70 participants, with a female predominance of 60% (n = 42). The majority were Saudi nationals (90%, n = 63), with a small proportion of non-Saudi participants (10%, n = 7). About 18.57% of the patients were newly diagnosed, and 81.43% were known cases of DM. Most participants did not have comorbidities (86.8%, n = 59), while a few had celiac disease (7.4%, n = 5) or other conditions (5.7%, n = 4), including atopic dermatitis, bronchial asthma, psoriasis, and short stature. The mean height and weight of the participants were 120 cm (SD = 19.5) and 28.7 kg (SD = 13.3), respectively. Additionally, 56.14% (n = 32) of the known cases had received DM education. The average age at DM onset was 6.9 years (SD = 3.3). The mean duration of hospital stay was 3.31 days (SD = 1.8), and the average frequency of diabetic ketoacidosis (DKA) was 2.1 times (SD = 2.4). A small percentage of participants experienced mental obtundation (3.9%, n = 2) as shown in Table 1.

**Table 1:** Demographic and clinical characteristics DM: Diabetes mellitus; SD: Standard deviation.

Variables		N (%)
Gender	Male	28 (40.0%)
	Female	42 (60.0%)
Nationality	Saudi	63 (90.0%)
	Non-Saudi	7 (10.0%)
Comorbidities	None	59 (86.8%)
	Celiac disease	5 (7.4%)
	Others	4 (5.7%)
Height, cm, Mean (SD)		120 (19.5)
Weight, kg, Mean (SD)		28.7 (13.3)
DM diagnosis	Newly diagnosed	13 (18.57%)
	Known cases	57 (81.43%)
	Missing	1 (1.4%)
Received DM education (% of known cases)	Yes	32 (56.14%)
	No	9 (15.79%)
	Missing	16 (28.07%)
Times of education visits, Mean (SD)		1.93 (0.9)
Age of DM onset, years, Mean (SD)		6.9 (3.3)

Medication and insulin therapy

All received medications were administered in an in-patient setting. A significant proportion of participants were on insulin therapy (97.1%, n = 66). Insulin types included glargine (84.3%, n = 59), aspart (84.3%, n = 59), glulisine (11.4%, n = 8), and regular insulin (8.6%, n = 6). The average insulin doses were as follows: glargine 15.3 IU/day, aspart 8.1 IU/8h, glulisine 10.2 IU/8h, and regular 8.2 IU/day. The total daily insulin dose averaged 39.4 IU (SD = 21.2) as shown in Table 2.

**Table 2:** Medications and insulin therapy SD: Standard deviation.

Variables		N (%)
Duration of hospital stay	Mean (SD)	3.31 (1.8)
DKA frequency	Mean (SD)	2.1 (2.4)
Mental obtundation		2 (3.9%)
Medications	Insulin	66 (97.1%)
	Insulin + Glucagon	2 (2.9%)
Type of insulin	Glargine	59 (84.3%)
	Aspart	59 (84.3%)
	Glulisine	8 (11.4%)
	Regular	6 (8.6%)
Dose of insulin, Mean (SD)	Glargine (IU/day)	15.3 (9.8)
	Aspart (IU/8h)	8.1 (4.4)
	Glulisine (IU/8h)	10.2 (1.83)
	Regular (IU/day)	8.2 (3.4)
Total daily dose, Mean (SD)		39.4 (21.2)
Long-acting dose, Mean (SD)		15.3 (9.8)
Missed insulin dose per week	1 time	6 (8.6%)
	2 times	1 (1.4%)
	3 times	1 (1.4%)
	None	8 (11.4%)

Biochemical parameters

On admission, biochemical analysis showed average levels of sodium at 136 mmol/L (SD = 5.6), potassium at 4.2 mmol/L (SD = 0.6), phosphate at 1.4 mmol/L (SD = 0.9), chloride at 100 mmol/L (SD = 9.4), thyroid-stimulating hormone (TSH) at 1.8 μIU/mL (SD = 1.7), free thyroxine (FT4) at 13.6 pmol/L (SD = 4.8), HbA1c at 11.8% (SD = 2.3), lactic acid at 2.9 mmol/L (SD = 0.7), and blood pH at 7.1 (SD = 0.15) as shown in Table 3.

**Table 3:** Laboratory findings on admission

Variables	Mean (SD)
Sodium	136 (5.6)
Potassium	4.2 (0.6)
Phosphate	1.4 (0.9)
Chloride	100 (9.4)
Thyroid-stimulating hormone	1.8 (1.7)
Free thyroxine	13.6 (4.8)
HbA1c	11.8 (2.3)
Lactic acid	2.9 (0.7)
Blood pH	7.1 (0.15)

Patient outcomes

The recovery rate was high, with 95.7% (n = 67) of participants recovered. A significant proportion (80%, n = 56) did not experience any complications. There were no reported deaths. Patient statuses varied, with 44.3% (n = 31) admitted to a ward and 12.9% (n = 9) requiring ICU admission due to severe metabolic acidosis, altered mental status, hemodynamic instability, or severe hypoglycemia. Among known cases with DM, 31.57% (n = 18) of patients resolved and discharged from ED (Table 4).

**Table 4:** Patients’ outcomes ICU: Intensive care unit.

Variables		N (%)
Recovery		67 (95.7%)
Complications	No	56 (80%)
	Not reported	12 (17.1%)
Death		0 (0.0%)
Patient status	Admitted to ward	31 (44.3%)
	Admitted to ICU	9 (12.9%)
	Resolved and discharged from ED (% of known cases)	18 (31.57%)

Comparison between DKA among newly diagnosed patients and those with prior diagnosis of DM

Notably, there was a significant difference in weight between the two groups, with newly diagnosed patients averaging 20.6 kg compared to 30.3 kg in known cases of DM, a statistically significant finding (p = 0.016). The insulin dosages that were followed as per hospital protocol differed significantly between the groups. The dose of glargine (IU/day) was 9.10 in newly diagnosed patients and 17.02 in known cases (p = 0.004), while the aspart (IU/8h) dose was 5.25 and 8.73, respectively (p = 0.008). Furthermore, the total daily insulin dose also showed a significant difference, being 22.5 IU in newly diagnosed patients compared to 42.7 IU in established cases (p = 0.002).

The study also found a significant difference in patient status, with newly diagnosed patients being more likely to be admitted to a ward (23.1% vs 49.1%, p = 0.04). The average education session for known cases of DM was 1.84 (0.88). Other parameters, including gender distribution, nationality, comorbidities, DKA frequency, and biochemical markers like HbA1c, lactic acid, and electrolytes, did not show significant differences between the two groups (Table 5).

**Table 5:** Comparison between DKA among newly diagnosed patients and those with a prior diagnosis of DM ICU: Intensive care unit; DKA: Diabetic ketoacidosis; DM: Diabetes mellitus.

Variables		DKA in newly diagnosed DM (n = 13)	DKA in known cases of DM (n = 57)	P-value
Gender	Male	7 (53.9%)	21 (36.8%)	0.279
	Female	6 (46.1%)	36 (61.4%)	
Nationality	Saudi	10 (76.9%)	52 (91.2%)	0.086
	Others	3 (23.1%)	4 (7.02%)	
Comorbidities	None	10 (76.9%)	49 (86.0%)	0.431
	Celiac disease	2 (15.4%)	3 (5.3%)	
	Others	1 (7.7%)	3 (5.3%)	
Weight		20.6 (7.97)	30.3 (13.7)	0.016
Height		114 (20.5)	121 (19.2)	0.244
DKA episode duration, hours		17.2 (8.47)	16.2 (7.59)	0.837
Hospital stays		3.42 (1.51)	3.13 (1.81)	0.616
Type of insulin	Glargine	12 (92.3%)	53 (93.0%)	0.932
	Aspart	12 (92.3%)	47 (82.4%)	0.378
	Glulisine	0 (0.0%)	8 (14.0%)	0.487
	Regular	1 (7.7%)	5 (8.7%)	0.900
Dose of insulin	Glargine (IU/day)	9.10 (5.30)	17.02 (10.06)	0.004
	Aspart (IU/8h)	5.25 (2.75)	8.73 (4.49)	0.008
Total daily dose		22.5 (12.4)	42.7 (20.9)	0.002
Doses with males		13.0 (7.99)	21.1 (12.6)	0.048
Long-acting dose		9.09 (5.30)	17.0 (10.1)	0.004
HbA1c		10.97 (1.50)	11.97 (2.43)	0.282
Lactic acid		3.36 (0.58)	2.55 (0.60)	0.134
Blood PH		7.07 (0.17)	7.08 (0.15)	0.791
Sodium		136.0 (6.26)	136.0 (5.55)	0.839
Potassium		4.58 (0.79)	4.28 (0.61)	0.431
Chloride		104.0 (9.81)	97.3 (8.48)	0.255
Patient status	Admitted to award	3 (23.1%)	28 (49.1%)	0.040
	Admitted to ICU	3 (23.1%)	6 (10.5%)	

Factors associated with ICU admission

In terms of the risk factors of ICU admission, the study identified weight as a significant predictor (OR = 1.26, 95% CI: 1.05 to 1.5; p = 0.011). Other variables, such as gender (male vs female), height, receipt of DM education, frequency of DKA episodes, total daily insulin doses, and whether the patient was newly diagnosed with DM did not demonstrate statistical significance in predicting the risk of DKA (Table 6).

**Table 6:** Factors associated with ICU admission ICU: Intensive care unit.

Risk factors		Odds ratio	95% confidence interval		P-value
			Lower	Upper	
Gender	Male vs Female	13.64	0.92	201.27	0.057
Weight		1.26	1.05	1.5	0.011
Height		0.93	0.84	1.03	0.154
Received DM education	No vs Yes	0.63	0.07	8.43	0.732
DKA frequency		0.17	0.02	1.39	0.098
Total daily doses		0.97	0.88	1.08	0.611
Newly diagnosed DM	Yes vs No	2.99	0.17	51.68	0.45

## Discussion

Interpretation of findings

The study's demographic findings reveal a predominance of female participants and a high representation of Saudi nationals, reflecting the population distribution of the region [13]. Similar to our findings, Almalki et al. showed a female predominance among pediatric patients with T1DM (61.5%) [12]. The low prevalence of comorbidities among the participants, primarily celiac disease, is noteworthy. The incidence of celiac disease in our population is comparable to the reported incidence in the literature [14-16]. It was reported that approximately 1%-16% of the T1DM patients had celiac disease compared to 0.3%-1% in the general population [14-16]. It is characterized by symptoms like gastrointestinal issues, malnutrition, and skin conditions [14-16]. Patients with both T1DM and celiac disease may experience inconsistent blood glucose levels, hypoglycemia, and glycemic deterioration, and a gluten-free diet can mitigate these symptoms in symptomatic children [17]. Diagnosis typically involves a biopsy, especially for asymptomatic children, although some may start a gluten-free diet based on high antibody titers and symptoms, with genetic screening being useful to confirm celiac disease risk [18,19]. In addition, the relatively low incidence of comorbidities in our population could be attributed to the shorter duration of the disease in our cohort.

Upon diagnosis, children and adolescents with diabetes, along with their families, should receive structured diabetes self-management education, proven more effective than unstructured teaching. This program covers fundamental aspects like diabetes pathogenesis, insulin types, and dosing, as well as managing ketosis and hypoglycemia [20]. In our study, the majority of the patients received DM education. Education can occur either in an in-patient setting post-medical stabilization or in an outpatient setting because studies have found the latter to be equally effective and more cost-efficient [21]. In-patient education might entail more aggressive insulin dosing compared to outpatient programs [22]. In our study, only 56.14% of known diabetic patients have documented diabetes education. As per hospital protocol, everybody should have diabetes education at diagnosis, so the lack of documented diabetes education sessions could reflect a red flag regarding the quality of the education. Besides, for those who have documented education, some of the details are lacking, such as sick day management and glucagon use for hypoglycemia, as well as carb counting, which are essential elements in T1DM education.

Insulin treatment should begin soon after a T1DM diagnosis and within six hours if ketosis is present to prevent DKA [23,24]. Post-DKA children often exhibit lower insulin sensitivity, necessitating higher initial insulin doses compared to those without DKA [25]. Generally, total insulin doses start at 0.5-0.75 U/kg/day at T1DM onset, with daily adjustments to maintain target blood glucose levels [20]. Obese or pubertal children usually require more insulin due to increased insulin resistance [26], while younger children need less, given their lower body weight, higher insulin sensitivity, and hypoglycemia risk. In very young children, total daily doses may be less than 0.5 U/kg/day, and diluted rapid-acting insulin analogs may be necessary for accurate dosing. Some institutions use U10 insulin (10 U/mL) instead of the standard U100 for very young children, although this is not needed with pump therapy, which is rarely started at the time of insulin initiation [27]. Our findings highlight the critical role of insulin in managing pediatric T1DM. The widespread use of insulin and varying types, notably glargine and aspart, is consistent with current pediatric diabetes management guidelines emphasizing the importance of insulin therapy [28]. The data on missed insulin doses highlight the challenge of adherence in pediatric diabetes, a well-documented issue that can significantly impact disease control and management outcomes [29].

The biochemical parameters, including average levels of sodium, potassium, phosphate, chloride, TSH, FT4, HbA1c, lactic acid, and blood pH, were within expected ranges for pediatric T1DM patients, indicating effective management of acute episodes and overall metabolic control. The high recovery rate (95.7%) and the low incidence of complications suggest successful acute management strategies in this cohort. Our findings showed that the mean duration of hospital stay was 3.31 days, which corroborates with established trends in pediatric T1DM, where an early onset often necessitates acute hospital care, particularly in cases complicated by DKA. Similar to our findings, Almalki et al. reported that the hospital stay duration among children with T1DM and DKA was 4.6 ± 3.3 days [12]. However, the fact that a notable percentage required ICU admission (12.9%) underscores the severity that DKA can present in pediatric T1DM patients. These findings align with other studies emphasizing the need for aggressive management in pediatric DKA to prevent complications and ensure rapid recovery [25,30]. Likewise, Almalki et al. demonstrated that the proportion of ICU admission was 19.3% [12]. The absence of mortality in our study stands out remarkably, especially when compared to other Saudi Arabian studies reporting mortality rates of 2.9%, 4.1%, and 3.5% [31]. This could be primarily attributed to the swift medical response in identifying DKA episodes at our facility. Continuous diabetes education emphasizing adherence to medical regimens, combined with addressing social and cultural factors leading to DKA and ensuring prompt medical care, can significantly decrease the incidence of DKA episodes.

There was a difference in insulin dosages required for children with newly diagnosed T1DM compared to those with a prior diagnosis. Newly diagnosed patients required lower doses of insulin, including glargine and aspart, aligning with studies like Almalki et al., which noted similar trends in insulin requirements in the initial phase post-diagnosis [12]. Moreover, the study highlighted a notable difference in patient status post-ER visit, with a higher discharge rate among newly diagnosed patients, underscoring the potential effectiveness of early and aggressive treatment strategies in newly diagnosed cases [28]. Additionally, weight emerged as a significant risk factor for DKA, which is consistent with findings from other studies indicating that higher body weight in pediatric patients correlates with increased DKA risk [8]. Contrary to some existing literature, factors such as gender, height, and receipt of DM education did not show a significant association with DKA risk in this study [8]. In known cases of patients with DM, the number of education sessions is missing from the chart, which could reflect the lack of efficient education for these patients; therefore, further studies are required to evaluate the quality of DM education and the parents’ perception in this group of population.

Clinical implications and future directions

The findings underscore the importance of tailored insulin therapy for children with newly diagnosed T1DM and highlight the need for vigilant monitoring of body weight as a part of comprehensive diabetes management. Additionally, research into the efficacy of targeted weight management programs as a part of diabetes care in children would be beneficial.

Limitations

This study's limitations include its retrospective design and the single-center setting, which may limit the generalizability of the findings. Additionally, the reliance on chart reviews may have introduced information bias. Another limitation was that this study did not reach the required sample size; therefore, further studies with larger sample sizes are required.

## Conclusions

This study highlights the importance of tailoring treatment approaches to meet the specific needs of children newly diagnosed with T1DM and DKA. It emphasizes the critical role of body weight as a risk factor for DKA and advocates for comprehensive management strategies that go beyond glucose monitoring to include lifestyle and dietary adjustments. The findings reveal that with prompt and effective emergency response, children with DKA can recover quickly and experience minimal complications, highlighting the potential for positive outcomes through early intervention. The study suggests a need for further research into the impact of diabetes education, community engagement, and culturally appropriate care on improving the lives of pediatric patients with T1DM.

## References

[1] Rivetti G, Hursh BE, Giudice EMD, Marzuillo P (2023). Acute and chronic kidney complications in children with type 1 diabetes mellitus. Pediatr Nephrol.

[2] EL-Mohandes N, Yee G, Bhutta BS, Huecker MR (2024). Pediatric diabetic ketoacidosis. https://pubmed.ncbi.nlm.nih.gov/29262031/.

[3] (2017). IDF Diabetes Atlas, Eighth edition. IDF Diabetes Atlas. Eighth edition 2017.

[4] Usher-Smith JA, Thompson M, Ercole A, Walter FM (2012). Variation between countries in the frequency of diabetic ketoacidosis at first presentation of type 1 diabetes in children: a systematic review. Diabetologia.

[5] Ahmed AM, Al-Maghamsi M, Al-Harbi AM, Eid IM, Baghdadi HH, Habeb AM (2016). Reduced frequency and severity of ketoacidosis at diagnosis of childhood type 1 diabetes in Northwest Saudi Arabia. J Pediatr Endocrinol Metab.

[6] Albishi LA, Altoonisi MM, Alblewi SM (2017). Clinical demographic patterns of type 1 diabetes in Saudi children in Tabuk City, 2000-2010. J Diabetes Mellit.

[7] Al-Hayek AA, Robert AA, Braham RB, Turki AS, Al-Sabaan FS (2015). Frequency and associated risk factors of recurrent diabetic ketoacidosis among Saudi adolescents with type 1 diabetes mellitus. Saudi Med J.

[8] Usher-Smith JA, Thompson MJ, Sharp SJ, Walter FM (2011). Factors associated with the presence of diabetic ketoacidosis at diagnosis of diabetes in children and young adults: a systematic review. BMJ.

[9] Hill-Briggs F, Adler NE, Berkowitz SA (2020). Social determinants of health and diabetes: a scientific review. Diabetes Care.

[10] Rodacki M, Pereira JR, de Oliveira AMN (2007). Ethnicity and young age influence the frequency of diabetic ketoacidosis at the onset of type 1 diabetes. Diabetes Res Clin Pract.

[11] Glaser N, Fritsch M, Priyambada L (2022). ISPAD clinical practice consensus guidelines 2022: diabetic ketoacidosis and hyperglycemic hyperosmolar state. Pediatr Diabetes.

[12] Almalki MH, Buhary BM, Khan SA, Almaghamsi A, Alshahrani F (2016). Clinical and biochemical characteristics of diabetes ketoacidosis in a tertiary hospital in Riyadh. Clin Med Insights Endocrinol Diabetes.

[13] (2024). Saudi Census 2022. https://portal.saudicensus.sa/portal.

[14] Craig ME, Prinz N, Boyle CT (2017). Prevalence of celiac disease in 52,721 youth with type 1 diabetes: international comparison across three continents. Diabetes Care.

[15] Rewers M, Liu E, Simmons J, Redondo MJ, Hoffenberg EJ (2004). Celiac disease associated with type 1 diabetes mellitus. Endocrinol Metab Clin North Am.

[16] Pham-Short A, Donaghue KC, Ambler G, Phelan H, Twigg S, Craig ME (2015). Screening for celiac disease in type 1 diabetes: a systematic review. Pediatrics.

[17] Abid N, McGlone O, Cardwell C, McCallion W, Carson D (2011). Clinical and metabolic effects of gluten free diet in children with type 1 diabetes and coeliac disease. Pediatr Diabetes.

[18] Rubio-Tapia A, Hill ID, Kelly CP, Calderwood AH, Murray JA (2013). ACG clinical guidelines: diagnosis and management of celiac disease. Am J Gastroenterol.

[19] Husby S, Koletzko S, Korponay-Szabó IR (2012). European society for pediatric gastroenterology, hepatology, and nutrition guidelines for the diagnosis of coeliac disease. J Pediatr Gastroenterol Nutr.

[20] (2023). 2011 Global IDF/ISPAD Guideline for Diabetes in Childhood and Adolescence. https://www.ispad.org/page/idfispadguidenews/2011-Global-IDFISPAD-Guideline-for-Diabetes-in-Childhood-and-Adolescen.htm.

[21] Jasinski CF, Rodriguez-Monguio R, Tonyushkina K, Allen H (2013). Healthcare cost of type 1 diabetes mellitus in new-onset children in a hospital compared to an outpatient setting. BMC Pediatr.

[22] Rewers M, Pihoker C, Donaghue K, Hanas R, Swift P, Klingensmith GJ (2007). Assessment and monitoring of glycemic control in children and adolescents with diabetes. Pediatr Diabetes.

[23] Lévy-Marchal C, Patterson CC, Green A (2001). Geographical variation of presentation at diagnosis of type I diabetes in children: the EURODIAB study. European and dibetes. Diabetologia.

[24] Rewers A, Klingensmith G, Davis C (2008). Presence of diabetic ketoacidosis at diagnosis of diabetes mellitus in youth: the search for diabetes in youth study. Pediatrics.

[25] Kostopoulou E, Sinopidis X, Fouzas S, Gkentzi D, Dassios T, Roupakias S, Dimitriou G (2023). Diabetic ketoacidosis in children and adolescents; diagnostic and therapeutic pitfalls. Diagnostics (Basel).

[26] Liu LL, Lawrence JM, Davis C (2010). Prevalence of overweight and obesity in youth with diabetes in USA: the SEARCH for diabetes in youth study. Pediatr Diabetes.

[27] Malik FS, Taplin CE (2014). Insulin therapy in children and adolescents with type 1 diabetes. Paediatr Drugs.

[28] Silver B, Ramaiya K, Andrew SB (2018). EADSG guidelines: insulin therapy in diabetes. Diabetes Ther.

[29] Jaser SS, Datye KA (2016). Frequency of missed insulin boluses in type 1 diabetes and its impact on diabetes control. Diabetes Technol Ther.

[30] Rosenbloom AL (2010). The management of diabetic ketoacidosis in children. Diabetes Ther.

[31] Al-Rubeaan KA, Aftab SA, Alotaibi MS, Alghamdi AA, Rafiullah MR (2011). Clinico-laboratory characteristics of diabetic keto acidosis in adults in a tertiary hospital in Saudi Arabia. Eur Rev Med Pharmacol Sci.

